# Astrocyte Changes in the Prefrontal Cortex From Aged Non-suicidal Depressed Patients

**DOI:** 10.3389/fncel.2019.00503

**Published:** 2019-11-12

**Authors:** Xin-Rui Qi, Willem Kamphuis, Ling Shan

**Affiliations:** ^1^Center for Translational Neurodegeneration and Regenerative Therapy, Shanghai Tenth People’s Hospital Affiliated to Tongji University School of Medicine, Shanghai, China; ^2^Netherlands Institute for Neuroscience, Royal Netherlands Academy of Arts and Sciences (KNAW), Amsterdam, Netherlands

**Keywords:** anterior cingulate cortex, bipolar disorder, diurnal rhythm, dorsolateral prefrontal cortex, glial fibrillary acidic protein, major depressive disorder

## Abstract

Glia alterations in the anterior cingulate cortex (ACC) and dorsolateral prefrontal cortex (DLPFC) have been postulated to play an important role in the pathophysiology of psychiatric disorders. Astroglia is the most abundant type of glial cells in the central nervous system. The expression levels of astrocyte markers (glial fibrillary acidic protein (GFAP), synemin-α, synemin-β, vimentin, nestin) in isolated gray matter from postmortem ACC and DLPFC were determined to investigate the possible involvement of astrocytes in depression. Donors were aged non-suicidal subjects with bipolar disorder (BPD) or major depressive disorder (MDD), and matched controls. GFAP mRNA levels were significantly increased in the ACC of BPD patients. However, GFAP immunohistochemistry showed that the area fraction of GFAP immunoreactive astrocytes was decreased in the ACC of BPD patients, while there were no changes in the cell density and integrated optical density (IOD), indicating that there might be a reduction of GFAP-positive astrocyte processes and remodeling of the astrocyte network in BPD. Furthermore, in controls, DLPFC GFAP mRNA levels were significantly lower with a time of death at daytime (08:01–20:00 h) compared to nighttime (20:01–08:00 h). In depression, such a diurnal pattern was not present. These findings in BPD and MDD subjects warrant further studies given the crucial roles of astrocytes in the central nervous system.

## Introduction

Mood disorders, such as bipolar disorder (BPD) and major depressive disorder (MDD), are common psychiatric illnesses (Ferrari et al., 2013; Bauer et al., 2018). The exact pathophysiological mechanisms underlying BPD and MDD remain largely unclear but are likely to be part of a complex interplay of genetic, developmental, and environmental factors. Based on neuroimaging studies, infarct locations in human, and lesion experiments in animal models, the dorsolateral prefrontal cortex (DLPFC) and anterior cingulate cortex (ACC) have been repeatedly implicated in the modulation of emotional behavior (Drevets et al., 2008a,b). Both functional changes, e.g., altered glucose metabolism and blood flow, and structural abnormalities, e.g., decreased gray matter volume of DLPFC or ACC, have been linked to the pathophysiology of mood disorders (Rodríguez-Cano et al., 2014; Orem et al., 2019; Yang et al., 2019).

Subtle changes in the density and size of cortical neurons have been reported in depression (Harrison et al., 2018). However, the changes to glial cells are more pronounced, including reduced cell density and altered morphology in the prefrontal brain regions of patients with mood disorders, i.e., in the ACC or in the DLPFC (Ongür et al., 1998; Cotter et al., 2002; Rajkowska and Stockmeier, 2013). Astrocytes are abundant throughout the human brain and are involved in many aspects of brain functions, such as glutamate neurotransmission and neuroinflammation (Wang et al., 2017). Interestingly, both of these were found to be abnormal or impaired in depression (Yirmiya et al., 2015; Wang et al., 2017).

In order to get a better insight into the possible involvement of astrocytes in the pathology of depression, we performed a quantitative real-time polymerase chain reaction (qRT-PCR) on gray matter isolated from freshly-frozen postmortem ACC and DLPFC of a well-characterized mood disorder cohort (aged non-suicidal mood disorder patients). We assessed the transcript levels of five intermediate filament encoding genes of astrocytes: glial fibrillary acidic protein (GFAP), vimentin, synemin-α, synemin-β, and nestin. Gene expression levels were related to the clock time of death of the subjects to check whether the diurnal pattern of gene expression was disrupted. Subsequently, immunohistochemistry on GFAP was performed to determine the cell density of GFAP-immunoreactive (ir) astrocytes as well as the integrated optical density (IOD) and area fraction covered by GFAP-ir astrocytic cell body and processes in the human ACC.

## Materials and Methods

### Subjects

Frozen postmortem brain material and paraffin-embedded ACC sections were obtained from the Netherlands Brain Bank (NBB), along with informed written consent from the patients or their next of kin for the autopsy and use of brain material and the use of their clinical files for research purposes. The DLPFC was obtained from 14 patients clinically diagnosed with a mood disorder, either (BPD, *n* = 9) or (MDD, *n* = 5), and from 14 matched controls without a psychiatric or neurological disease. The ACC was obtained from 12 patients with BPD (*n* = 7) or MDD (*n* = 5) and from 12 matched controls. BPD patients and their controls, as well as MDD patients and their controls, were pair-matched for sex, age, postmortem delay (PMD), clock time and month of death, cerebrospinal fluid (CSF)—pH and brain weight. DSM-IV criteria were used for the extensively described clinical diagnosis of MDD or BPD during lifetime. The criteria for the presence and severity of symptoms of either MDD or BPD were confirmed, and other psychiatric and neurological disorders were systematically excluded by three highly experienced psychiatrists (Drs. W.J.G. Hoogendijk, E. Vermette or G. Meynen). The absence of neuropathological changes, both in the patients with mood disorders and in the controls, was confirmed by systematic neuropathological investigation (van de Nes et al., 1998). As some of our previous studies have shown that depressed patients who died by suicide have different neurochemical profiles compared to non-suicidal patients (Zhao et al., 2016, 2018), it is of importance to note that none of the patients with mood disorders were suicide victims. The age (mean ± SEM, years) was 74.6 ± 3.0 for controls in the DLPFC studies, 75.1 ± 2.4 for BPD subjects, and 68.8 ± 7.6 for MDD subjects. For the ACC studies, this was 79.5 ± 3.0 for controls, 78.9 ± 3.6 for BPD subjects, and 68.8 ± 7.6 for MDD subjects. Further details about the diagnostic procedures and methods for collecting information on the subjects have been described before Qi et al. (2015) and are provided in Supplementary Tables S1, S2.

### Tissue Dissection and Gray Matter Collection

Cryostat sections of 50 μm were obtained from snap-frozen postmortem cortex samples. Gray matter areas were identified macroscopically and confirmed by Nissl staining in alternating sections. The dissection was performed with the use of pre-chilled scalpels. Gray matter was collected into pre-chilled 2 ml tubes and immediately put on dry ice. All the procedures were performed at −18°C. For each sample, around 50 mg of gray matter was collected.

### RNA Isolation and cDNA Synthesis

Total RNA was isolated from the collected gray matter according to the procedure described by Wang et al. (2008). For each sample, 1 μg total RNA was used for the synthesis of cDNA. DNase treatment of RNA samples was performed prior to reverse transcription by reverse transcriptase Superscript II RT according to the manufacturer’s protocol (Invitrogen Life Technologies).

### Target Genes Chosen for Our Study

For astrocytes, intermediate filament proteins including GFAP (the canonical markers for astrocytes; Eng et al., 2000), vimentin (immature and reactive astroglia; Pekny and Pekna, 2004), nestin (immature astroglia and reactive astroglia; Hol and Pekny, 2015), synemin-α, synemin-β (reactive astroglia; Jing et al., 2007) were chosen as markers. Primers designed for the target genes are shown in Supplementary Table S5.

### Quantitative Real-Time PCR

QPCR reactions and calculations have been described in detail before (Qi et al., 2013, 2018). The absolute amount of target genes was calculated by 10^10^ × E^−Ct^ (*E* = 10^−(1/slope)^). The normalization strategy provided by Vandesompele was used to select a number of stably expressed reference genes to provide a reliable normalization factor to compensate for the sampling differences such as RNA quantity and quality (Vandesompele et al., 2002). The transcript levels of seven potential normalization candidates were determined: [glyceraldehyde-3-phosphate dehydrogenase (GAPDH), actin-β (ACTβ), hypoxanthine phosphoribosyltransferase 1 (HPRT1), ubiquitin C (UBC), tubulin-α (TUBα), tubulin-β4 (TUBβ4), and hydroxymethylbilane synthase (HMBS)]. Following geNorm analysis, the following genes were selected: ACTβ, HPRT1, UBC, TUBα, TUBβ4 for the ACC samples and GAPDH, ACTβ, HMBS, HPRT1, TUBα, TUBβ4 for DLPFC samples. The absolute amount of the transcript obtained was divided by the geomean of the absolute amounts of the reference genes to get the normalized relative value (mRNA relative value in the figures). These values were used for the final statistical analysis.

### Immunohistochemistry for GFAP in the Paraffin Human Brain Sections

The immunostaining protocol was performed as described in our previous study (Shan et al., 2012a). After deparaffinization and rehydration, antigenicity was retrieved by heating sections in TBS, pH 7.6, in a water bath at 90°C for 20 min. To block background staining, sections were pre-incubated in 5% TBS-milk, followed by incubation with polyclonal rabbit anti-GFAP antibody (Dako, Denmark). The antibody specificity has been previously tested (Middeldorp et al., 2010). The antibody was diluted in TBS buffer containing 0.5% (v/v) Triton-X 100, 0.25% (w/v) gelatin and 5% (w/v) milk (SUMI) and incubated with the sections at room temperature for 1 h, followed by overnight incubation at 4°C. After washing, the sections were incubated with biotin-labeled anti-rabbit-IgG (Vector Laboratories, Burlingame, CA, USA), followed by avidin-biotin complex (Vector Laboratories, Burlingame, CA, USA) at room temperature for 1 h. Three TBS washes were conducted between incubations. Finally, sections were stained with 3.3′-diaminobenzidine (DAB, Sigma) at 0.5 mg/ml in TBS, containing 0.23% (w/v) nickel-ammonium sulfate (Merck, Darmstadt, Germany) and 0.04% H_2_O_2_. The reaction was stopped by washing in TBS and sections were counterstained with hematoxylin for 50 s followed by rinsing under running tap water for 10 min. Sections were dehydrated, cleared in xylene and coverslipped using Entellan (Merck, Darmstadt, Germany). Omission of either primary or secondary antibody yielded no discernable immunostaining.

### Quantification of GFAP Immunohistochemistry

#### Cell Counting

The cell counting system consisted of a Zeiss Axioskop microscope (Zeiss, Germany) with neofluar objectives (Zeiss, Germany) and a motorized XYZ stage, Evolution MP color camera (Mediacybernetics, Rockville, MD, USA) in combination with the software Image-Pro 6.3 (Mediacybernetics, Rockville, MD, USA) plus home developed macros. The method of cell counting in the ACC was similar to what was described in our earlier work (Gao et al., 2013). Briefly, after an image was collected at low magnification (10× objective), the intact gray matter extending from the pia to the gray-white matter border was outlined. The outlined area was then divided into subfields by a macro for cell counting at 40× magnification. Randomly selected subfields were counted under a ×40 microscope objective, covering in total 45% of the manually outlined areas (45% of gray matter area was needed to get a proper estimate of cell density for the whole area after a pilot study). Cells double positive for GFAP and hematoxylin (GFAP-ir cells) were counted. The estimated GFAP-ir cell densities per cubic millimeter were calculated as the number of GFAP-ir cells divided by the measured area and thickness of the section (6 μm). All six layers were included.

#### Area Fraction Covered by GFAP-ir Astrocytic Cell Body and Processes

A black and white camera (Sony, Japan) was fitted on the microscope with a 20× objective in front. The light was carefully adjusted to make sure the same OD for unstained areas in all sections. Collected images were transformed into OD images by the use of a transformation curve. The gray matter with six layers in the ACC was delineated and OD values of the delineated area which were above 2.5 times of the background were considered as a positive signal, after a pilot study to determine this threshold value. All larger blood vessels were excluded from the area of interest. OD analysis was performed with the software Image-Pro 6.3 (Mediacybernetics) plus home-developed macros. The OD values for the GFAP signal and the area fraction covered by the GFAP signal were calculated. The integrated OD (IOD) was calculated by multiplying the OD of positive signals with the area fraction covered by the GFAP signal, as representing the total amount of protein (Gao et al., 2018).

All staining, counting and cell measurements were done on coded tissue, with the researchers doing this work blinded to the nature of the tissue. The same rater counted both experimental and control tissues in a randomized order.

### Statistical Analysis

Differences in both qPCR and immunohistochemical data were tested by the non-parametric Mann–Whitney *U*-test using SPSS (version 17.0, SPSS Incorporation) because some data were not normally distributed. The differences between BPD or MDD and their respective controls were analyzed in the ACC and DLPFC separately. For differences between daytime and nighttime expression levels (clock time of death for subjects: 08:01–20:00 h vs. 20:01–08:00 h), Mann–Whitney *U*-test was conducted within control group and mood disorder group, according to our previous study (Shan et al., 2012b). The presence of possible differences in confounding factors such as age, pH of the CSF, brain weight, and PMD was also tested by nonparametric Mann–Whitney *U*-test. For group differences in the clock time of death (circadian parameters), the Mardia-Watson-Wheeler test was used. Furthermore, possible differences in relation to ApoE genotype and Braak stage were computed by the Kolmogorov–Smirnov test. To evaluate correlations, Spearman’s correlation coefficient was determined. *P*-values of less than 0.05 were considered significant.

## Results

### Up-regulation of GFAP mRNA Expression Level and Reduced Area Fraction of GFAP-Immunoreactive Astrocyte in the ACC in Bipolar Disorder

**Figure 1:**
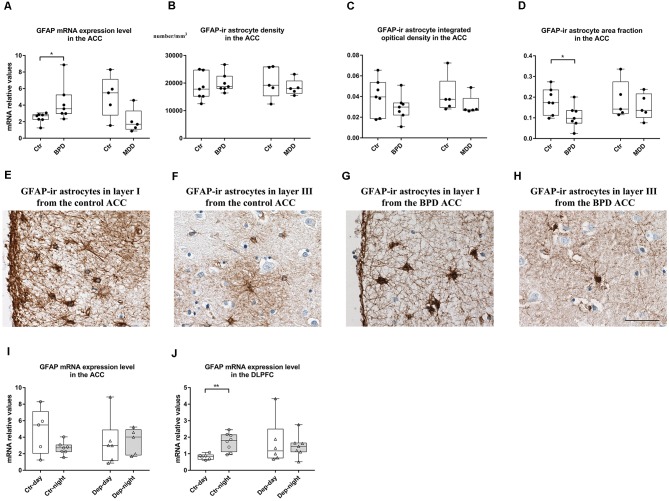
Changes in glial fibrillary acidic protein (GFAP) in the anterior cingulate cortex (ACC) and dorsolateral prefrontal cortex (DLPFC) of bipolar disorder (BPD) and major depressive disorder (MDD) patients. In the gray matter of the ACC, transcript levels of the GFAP were significantly increased in the BPD group, and a trend towards a decrease was observed in the MDD patients **(A)**. The density **(B)** and integrated optical density (IOD; **C**) of GFAP-immunoreactive (ir) positive astrocytes were stable in both BPD and MDD groups. The area fraction of GFAP-ir astrocytes was significantly decreased in the BPD group, and unaltered in the MDD group **(D)**. Representative images of GFAP-ir astrocytes in layer I **(E)** and layer III **(F)** from the ACC of a control subject (NBB No. 04-049) and in the layer I **(G)** and layer III **(H)** from the ACC of a BPD patient (NBB No. 02-014). Scale bar = 50 μm in **(H)**. In the ACC, GFAP mRNA expression did not show any day-night fluctuations in either control subjects or depressed patients **(I)**. In the DLPFC, GFAP mRNA expression was significantly decreased during daytime compared to that during nighttime in controls and these differences between daytime and nighttime were disrupted in mood disorder patients **(J)**. Boxplots showing the median, 25th-75th percentiles, the range of the parameters and individual data points. The four columns in **(A–D)** are data from BPD patients (BPD) and their matched controls (Ctr), as well as MDD patients (MDD) and their matched controls (Ctr). The four columns in **(I,J)** are data from control subjects with a clock time of death during the day (Ctr-day) and night (Ctr-night), as well as mood disorder patients with a clock time of death during the day (Dep-day) and night (Dep-night). *P < 0.05, **P < 0.01.

In the ACC, there was a statistically significant increase of mRNA expression level of GFAP in the patients with BPD compared to controls (*p* = 0.018), whereas a trend for a decrease was observed in the subjects with MDD compared to controls (*p* = 0.076, Figure 1A). Transcript levels of the other genes investigated (vimentin, synemin-α, synemin-β, nestin) were not statistically significant altered (*p* > 0.05, Supplementary Table S3). In addition, in control subjects, the mRNA expression level of GFAP was found to be significantly correlated with those of vimentin (Spearman’s rho = 0.692, *p* = 0.013), and synemin-β (Spearman’s rho = 0.888, *p* = 0.001). In BPD patients, there was a significant correlation between the mRNA expression level of GFAP with those of synemin-β (Spearman’s rho = 0.857, *p* = 0.014), and vimentin (Spearman’s rho = 0.893, *p* = 0.007). In MDD patients, on the other hand, there were no significant correlations (Supplementary Table S4).

In the GFAP staining, we found that GFAP-ir astrocytes were mostly situated in layer I with highly ramified processes, which were also distributed throughout other layers (II-VI) of the ACC (Supplementary Figure S1). Quantification of the staining revealed that there was no difference in the GFAP-ir astrocyte density in the gray matter in patients with BPD or MDD (*p* > 0.05, Figure 1B). However, the area fraction was significantly decreased (*p* = 0.035, Figure 1D) with no changes in the IOD (*p* = 0.225, Figure 1C) in the gray matter of the BPD patients. The area fraction and IOD of GFAP-ir astrocytes were stable in the MDD patients (*p* > 0.05, Figures 1C,D). Representative images from control subjects (Figures 1E,F) and BPD patients (Figures 1G,H) showed a clear reduction of GFAP positive branches and/or loss of immunoreactive somata in the gray matter of the ACC in BPD. Furthermore, in control subjects, the mRNA expression level of GFAP was found to be significantly correlated with the IOD of GFAP-ir astrocytes (Spearman’s rho = 0.650, *p* = 0.022). On the other hand, in the BPD and MDD cohorts, there were no significant correlations between GFAP mRNA levels and the IOD of GFAP-ir astrocytes (Supplementary Table S4).

When we compared the GFAP mRNA levels in the ACC between the control subjects with a clock time of death during daytime (*n* = 5, 08:01-20:00 h) and those with a clock time of death during nighttime (*n* = 7, 20:01–08:00 h), no significant differences were found (*p* = 0.167, Figure 1I). This was also the case for mood disorder patients (*n* = 6, 08:01–20:00 h vs. *n* = 5, 20:01–08:00 h, *p* = 0.465, Figure 1I).

There was no significant correlation between GFAP mRNA expression level, the density, area fraction and IOD of GFAP-ir astrocytes with age, PMD, CSF pH in either BPD or MDD group or control group (*p* > 0.05).

### Disrupted Day-Night Fluctuations of GFAP mRNA Level in the DLPFC in Depression

In the DLPFC, we found no statistically significant changes in the target gene mRNA expression levels in BPD or MDD patients compared to their respective controls (*p* > 0.10, Supplementary Table S3). However, when we analyzed the data between the control subjects with a clock time of death during daytime (*n* = 6, 08:01–20:00 h) and those with a clock time of death during nighttime (*n* = 8, 20:01–08:00 h), significantly lower GFAP mRNA level was observed during daytime (*p* = 0.005). In contrast, this reduction of GFAP mRNA expression level during daytime was absent in the mood disorder patients, comparing GFAP mRNA level from mood disorder patients with a clock time of death during daytime and those from the mood disorder patients with a clock time of death during nighttime (*n* = 6, 08:01–20:00 h vs. *n* = 7, 20:01–08:00 h; *p* = 0.775, Figure 1J).

## Discussion

In the present study, we reported a significant increase in the mRNA expression level of the astrocyte marker GFAP in the ACC of the BPD patients. Our immunohistochemical results in the ACC showed that the density and IOD of GFAP-ir cells were stable in the BPD, which suggested no alterations in GFAP positive cell number and GFAP protein expression. In contrast, the area fraction of GFAP-ir astrocytes was decreased, indicating that there was a reduction of the GFAP positive astrocyte branches and/or lower expression of GFAP staining on individual astrocyte cell bodies. Interestingly, in the same brain region, only a trend for a decrease in GFAP mRNA expression level was found in MDD patients, with no significant changes in the density, IOD, and area fraction of GFAP-ir astrocytes. Finally, a remarkable diurnal pattern of GFAP mRNA expression appeared to be present in the DLPFC of healthy controls, but not in mood disorder patients. Alterations of GFAP expression may reflect pathological regulation of astrocytes in neuronal function, survival, synaptogenesis, and neurotransmission (Halassa and Haydon, 2010). These results indicate that the altered function of astrocytes in the ACC and DLPFC might be involved in the pathophysiology of mood disorders.

While astrocyte abnormalities have previously been reported in BPD and MDD patients (Zhao et al., 2016; Wang et al., 2017; Giridharan et al., 2019), the changes in their size, density, area fraction as well as the gene expression of their specific markers in cortical gray matter of ACC and DLPFC remains inconclusive. Most investigators have chosen GFAP as specific markers for astrocytes, as it is the protein mostly associated with astrocytic functions. For example, the mRNA and protein expression levels of GFAP was down-regulated in depressed suicide victims (Nagy et al., 2015; Torres-Platas et al., 2016). Some other studies on GFAP protein or mRNA expression in the cortical gray matter have identified increased expression (Feresten et al., 2013), or no significant change (Webster et al., 2005; Dean et al., 2006). GFAP immunocytochemical investigations have also yielded inconsistent results (Miguel-Hidalgo et al., 2000; Davis et al., 2002; Williams et al., 2013; Vernon et al., 2014). The diversity of studies and variations in results due to non-standardized confounding factors, such as age or PMD, whose impact may change the marker profiles, make it difficult to thoroughly compare the studies and come to an overall conclusion.

In the ACC, elevation of GFAP mRNA level of aged non-suicidal BPD patients in our study is in good agreement with a previous study showing augmented mRNA and protein levels of GFAP in the frontal cortex of BPD patients (Rao et al., 2010). However, GFAP mRNA levels did not show any significant difference in the gray matter of ACC in another study (Webster et al., 2005). These disparities might be due to the difference in age of the patients between our study and Webster’s study as it has been reported that GFAP expression is significantly and positively correlated with age, with more pronounced changes in younger mood disorder patients (Si et al., 2004; Barley et al., 2009). The average age of the BPD patients in our cohort was 78.9 years, while in Webster’s study the average age of the BPD patients was 42.3 years (Webster et al., 2005). Another reason for the inconsistency might be suicide behaviors, as suicidality is a confounder for postmortem studies on depression (Zhao et al., 2019). For example, glutamatergic and GABAergic gene expression profiling in the prefrontal cortex (PFC) are completely different between suicide and non-suicide cases (Zhao et al., 2016, 2018). None of the depressed patients in our study died by suicide while 9/15 of the Webster cohort died by suicide (Webster et al., 2005). Additionally, there are no changes in the density of GFAP-ir astrocytes in the present study, which is consistent with previous findings (Williams et al., 2013), indicating the absence of astrocyte proliferation in the ACC of BPD patients. Decreased area fraction without any changes in the IOD (protein level index) of GFAP-ir astrocyte in the present study may suggest retraction of GFAP positive fibers comparable to the phenomenon seen in the astrocytes of supraoptic nucleus during initial suckling in lactating rats in relation to changes in oxytocin neuronal activity (Wang and Hatton, 2009; Hou et al., 2016). This reduction of GFAP-ir astrocyte fibers may be an important mechanism underlying the excitotoxicity of neurons in neurodegenerative diseases (Verkhratsky et al., 2017). The remodeling of the astrocyte network could also induce reduced astrocytic coverage of synapses (Poskanzer and Molofsky, 2018), which might be involved in aberrant neurotransmission or synaptic plasticity (Panatier et al., 2011). Therefore, the rearrangement of intermediate filaments such as GFAP in astrocytes may lead to dysfunctional astrocytes in aged non-suicidal BPD patients. Additionally, to the best of our knowledge, this is the first study revealing GFAP expression patterns in the gray matter of ACC of MDD patients. We found a trend towards a significant decrease in the ACC in MDD that might have been significant if we would have a larger sample size. In addition, we found that mRNA expression level of vimentin and synemin-β exhibited a significantly positive relationship with the mRNA expression level of GFAP in the control and the BPD patients but not in the MDD patients, which suggest that the connections between GFAP and vimentin or GFAP and synemin-β are stable in BPD but not in MDD. Only IOD of GFAP-ir astrocytes was found to be correlated with GFAP mRNA levels in control subjects but not in BPD nor MDD patients, suggesting that regulation of GFAP in astrocytes is impaired or at least abnormal in mood disorders.

In the DLPFC, several lines of evidence have shown that the density and area fraction of GFAP-ir astrocytes, as well as GFAP mRNA expression levels, are significantly decreased in MDD or BPD patients (Rajkowska and Stockmeier, 2013; Harrison et al., 2018). We did not find any significant differences in GFAP mRNA expression levels in the DLPFC of aged non-suicidal mood disorder patients, but this can be explained by observations showing that GFAP changes in the DLPFC can be age-dependent (Si et al., 2004). In the latter study, the reduction found in GFAP protein levels in depressed subjects was mainly based on the difference in depressed subjects younger than 60 years, while there was no significant difference in GFAP in patients older than 60 years, which seems to be in agreement with our results.

We showed that there is a clear day-night GFAP fluctuation in the DLPFC in control subjects, however, in the mood disorder patients this diurnal difference was absent. It has been reported that there is a circadian influence on expression patterns of quite a large number of genes, especially the clock genes in postmortem human cortex reviewed by microarray analysis (Li et al., 2013; Chen et al., 2016). However, these studies did not focus on astrocytes and, in fact, neurons were the focus of circadian rhythm studies. However, recently, the role of the astrocyte itself in regulating circadian rhythms and behaviors (Chi-Castañeda and Ortega, 2018), particularly the astrocytes in the suprachiasmatic nucleus (SCN), have been studied intensively (Lim et al., 2017; Tso et al., 2017; Brancaccio et al., 2019). Furthermore, an *in vitro* study showed that cultured cortical astrocytes from rat and mouse expressed circadian oscillators (Prolo et al., 2005), and ATP release from cortical astrocytes exhibited a circadian pattern, which is relying on functional clock genes expression such as *clock* and *period* (Marpegan et al., 2011). In addition, extracellular ATP content is associated with various brain functions, such as sleep (Jones, 2009), regulated by the PFC (Owens et al., 2000), which is frequently disturbed in the patients with psychiatric disorders in general (Wulff et al., 2012), and mood disorders in particular (Bagherzadeh-Azbari et al., 2019; Steardo et al., 2019). It should also be noted that GFAP expression reflects the neuronal activity state (Hajós, 2008); the observed GFAP expression fluctuation thus supports the existence of neuronal day-night activity rhythm in the human DLPFC. Indeed, neuronal morphological changes, such as in the dendritic structure and spine density of the pyramidal neurons in the rat PFC, have been shown to exhibit a circadian rhythm. For example, during the night, the active period of rats, the dendrites were longer and more complex (Perez-Cruz et al., 2009). The mechanisms responsible for generating and entraining the circadian rhythm in the prefrontal cortical astrocytes, and their relationship with the body’s pacemaker in the SCN, need further study.

Possible confounders in our study, such as age, PMD and CSF-pH, were well-matched between BPD/MDD patients and their respective controls (see Supplementary Table S2) and these factors will consequently not have affected our conclusions. Indeed, given the fact that very few female subjects were present in the current study (see Supplementary Table S2; one in MDD ACC/DLPFC materials, two in BPD ACC materials, three in BPD DLPFC materials), it was not possible to statistically evaluate possible sex differences. However, when we plotted our data, we found that the gene expression levels of males and females were intermingled in both the control and BPD/MDD groups, suggesting a sex difference is not present. There are, however, several limitations to the current study. First, our sample size was relatively small. Therefore, the observed alterations deserve further investigation in a study with a larger sample size. However, it should be kept in mind that clinically and neuropathologically well-documented postmortem material from aged depressed patients who did not die by suicide is difficult to come by. In the present study, we only studied intermediate filament proteins in astrocytes and some proteins such as vimentin are mainly expressed by blood vessels in the healthy adult brain. Therefore more functional or specific proteins expressed in astrocytes (i.e., connexin 30; Nielsen et al., 2017) in double-stained sections with GFAP should be included in future studies to gain a more complete overview of the astrocytic pathology in BPD and MDD. Additionally, not all astrocyte express detectable GFAP (Khakh and Sofroniew, 2015), which might have prevented us to expand our conclusion to a broader range of astrocytes, i.e., to non-GFAP-ir astrocytes. It should also be noted that a limitation of this study is that we did not use stereology for the analysis of the immunohistochemical GFAP staining. Care must be taken when interpreting our results as the BPD and MDD patients were treated with antidepressants, which might theoretically have affected the current results. However, the regulation of astrocyte-associated genes in the present study is unlikely to be the result of the chronic administration of antidepressants. Two mood disorder patients (NBB No. 02-014 and 99-188) were antidepressant free for the last 3 months (Supplementary Table S1) and their gene expression data fell well in the range of other mood disorder cases with antidepressant treatment. Nonetheless, future studies should assess patients consisting of mood disorders who are not on any antidepressant medication. Finally, the day-night difference of GFAP mRNA expression levels was determined in the pooled BPD and MDD patients, even though BPD and MDD are two different psychiatric entities in diagnostic terms. However, they share similarities with regard to their neurobiological underpinnings. For example, both BPD and MDD have a genetic variation in genes pertaining to the molecular circadian machinery such as CRY1 (rs2287161), NPAS2 (rs11123857), and VIPR2 (rs885861) genes (Soria et al., 2010). Disruptions in circadian rhythms have been reported in both MDD and BPD patients, as expressed by disrupted sleep/wake cycles (Bagherzadeh-Azbari et al., 2019; Steardo et al., 2019). In addition, a proportion of both MDD and BPD patients have increased hypothalamic-pituitary-adrenal axis activity, as shown by an increased number of corticotropin-releasing hormone (CRH)-expressing neurons and amount of CRH mRNA in the paraventricular nucleus (Bao et al., 2008; Wang et al., 2008), which is important in relation to day-night fluctuations. Additionally, none of the BPD patients in our study was in a manic phase. Thus, we pooled both BPD and MDD patients together in one mood disorder group and compared the day-night differences of GFAP mRNA expression level within this group. Still, the day-night fluctuations of GFAP mRNA and protein expression levels deserve further investigation in separate BPD and MDD cohorts with larger sample size.

## Conclusion

To summarize, specific GFAP dysregulation in the ACC and/or DLPFC are associated with aged and non-suicidal depression. The mechanism by which this deficit occurs is not known, but it may adversely influence the regulation of neuronal metabolism, communication, and activity. In addition, a remarkable diurnal pattern of the GFAP mRNA expression appeared to be present in the DLPFC of controls, but not in mood disorder patients. Future investigations will need to confirm the observed astrocytic abnormalities and dysfunction and characterize the determining underlying mechanism.

## Data Availability Statement

All datasets generated for this study are included in the article/Supplementary Material.

## Ethics Statement

The studies involving human participants were reviewed and approved by VU University medical center ethics committee. Written informed consent for tissue donation to this study was provided by the participants’ legal guardian/next of kin or the donors themselves.

## Author Contributions

X-RQ and LS conceived the project and wrote the manuscript. X-RQ performed the experiments. X-RQ and WK analyzed the data.

## Conflict of Interest

The authors declare that the research was conducted in the absence of any commercial or financial relationships that could be construed as a potential conflict of interest.
