# Supplementary material for: Astrocyte Changes in the Prefrontal Cortex From Aged Non-suicidal Depressed Patients
Source: Front Cell Neurosci. 2019 Nov 12;13:503. doi: 10.3389/fncel.2019.00503 (PMC6874137; doi:10.3389/fncel.2019.00503)
Supplement: Supplementary file 1 [file Data_Sheet_1.docx]

**Table S1. Clinico-pathological information of patients with mood disorders and control subjects.**

| **Number** | **NBB number** | **Group** | **Sex** | **Age (year)** | **PMD (hr:min)** | **Ventricular CSF pH ^1^** | **Brain weight (g)** | **Clock time at death (hh:mm)** | **RIN values**  **DLPFC/ACC** |
| --- | --- | --- | --- | --- | --- | --- | --- | --- | --- |
| D1 | 00-074 | BPD | M | 78 | 07:35 | 6.27 | 1 227 | 23:00 | 7.3/6.7 |
| D2 | 00-088 | BPD | M | 73 | 05:15 | 6.38 | 1 260 | 09:30 | 7.2/7.5 |
| D3 | 00-111 | BPD type I | M | 70 | 04:50 | 6.26 | 1 490 | 02:45 | 6.9/- |
| D4 | 02-014 | BPD type I | M | 68 | 16:46 | 6.64 | 1 424 | ND | 8.1/7.2 |
| D5 | 06-021 | BPD | M | 70 | 06:23 | 6.53 | 1 488 | 13:07 | 7.1/7.0 |
| D6 | 06-075 | BPD | F | 80 | 09:30 | 6.33 | 1 190 | 09:30 | 6.3/6.3 |
| D7 | 07-060 | BPD | M | 93 | 06:00 | 6.37 | 1 459 | 21:10 | -/7.7 |
| D8 | 07-076 | BPD | F | 79 | 07:25 | 6.26 | 1 231 | 03:10 | 7.2/- |
| D9 | 97-058 | BPD type II | F | 90 | 06:30 | ND | 1 143 | 10:15 | 7.4/7.1 |
| D10 | 99-118 | BPD | M | 68 | 05:55 | 6.82 | 1 204 | 23:15 | 7.7/- |
| D11 | 01-074 | MDD | M | 45 | 07:00 | 6.55 | 1 427 | 02:30 | 8.0/7.0 |
| D12 | 02-051 | MDD | M | 81 | 06:00 | 6.50 | 1 345 | 15:30 | 7.3/8.0 |
| D13 | 06-011 | MDD | F | 60 | 04:20 | ND | 1 080 | 16:10 | 8.9/8.1 |
| D14 | 06-026 | MDD | M | 70 | 07:15 | 6.50 | 1 415 | 08:00 | 8.4/7.9 |
| D15 | 07-033 | MDD | M | 88 | 06:37 | 6.26 | 1 225 | 21:15 | 7.3/7.0 |
| C1 | 00-067 | Control | M | 73 | 24:45 | ND | 1 267 | 00:01 | 7.8/- |
| C2 | 01-033 | Control | M | 75 | 06:20 | 6.18 | 1 180 | 06:10 | 7.3/- |
| C3 | 01-086 | Control | M | 88 | 07:00 | 6.84 | 1 398 | 03:00 | 8.1/- |
| C4 | 04-020 | Control | M | 96 | 05:23 | 6.70 | 1 204 | 13:27 | -/7.4 |
| C5 | 04-049 | Control | F | 77 | 08:20 | 6.48 | 1 312 | 07:55 | 6.1/5.9 |
| C6 | 04-057 | Control | F | 81 | 06:40 | 7.16 | 1 164 | 13:10 | 8.2/7.5 |
| C7 | 05-017 | Control | M | 87 | 10:20 | 6.32 | 1 356 | 04:00 | -/7.5 |
| C8 | 05-019 | Control | M | 74 | 05:00 | 6.70 | 1 125 | 02:00 | 8.3/8.1 |
| C9 | 05-034 | Control | M | 56 | 14:00 | 7.03 | 1 323 | 00:01 | 8.5/- |
| C10 | 05-044 | Control | M | 80 | 07:15 | 5.80 | 1 376 | 07:15 | 5.7/5.2 |
| C11 | 05-068 | Control | M | 56 | 09:15 | 6.54 | 1 553 | 04:45 | -/7.9 |
| C12 | 05-073 | Control | M | 87 | 06:05 | 6.96 | 1 568 | 08:05 | -/7.8 |
| C13 | 06-037 | Control | M | 66 | 07:45 | 6.70 | 1 590 | 17:45 | 7.8/- |
| C14 | 06-080 | Control | F | 89 | 06:25 | 6.46 | 1 210 | 21:30 | -/7.1 |
| C15 | 95-062 | Control | M | 80 | 04:30 | 6.22 | 1 400 | 14:30 | -/5.4 |
| C16 | 96-052 | Control | M | 73 | 09:10 | ND | 1 500 | 11:30 | -/8.5 |
| C17 | 97-039 | Control | M | 87 | 04:00 | 7.39 | 1 506 | 15:00 | 8.3/- |
| C18 | 97-043 | Control | M | 68 | 10:10 | 7.08 | 1 547 | 09:05 | -/8.5 |
| C19 | 97-143 | Control | M | 79 | 06:00 | 6.51 | 1 392 | 06:10 | -/8.6 |
| C20 | 97-156 | Control | F | 77 | 02:40 | 6.37 | 1 235 | 08:30 | 8.2/- |
| C21 | 98-006 | Control | M | 50 | 08:30 | 6.65 | 1 436 | 11:00 | 7.5/- |
| C22 | 99-111 | Control | F | 88 | 05:40 | 6.67 | 1 054 | 03:05 | 6.9/- |

**Table S1 Continued**

| **Number** | **NBB number** | **Medication in the last 3 months** | **Died during**  **depressive episode** | **Brain Region** | **Family history** | **Suicide** | **Cause of death** |
| --- | --- | --- | --- | --- | --- | --- | --- |
| D1 | 00-074 | BZD | Yes | L ACC, L DLPFC | Yes | No | Metastases of colon cancer |
| D2 | 00-088 | Mo | Yes | L ACC, R DLPFC | Yes | No | Dehydration |
| D3 | 00-111 | Mo, ZUC | Yes | L DLPFC | Yes | No | Cardiac arrest, ileus |
| D4 | 02-014 | None | Yes | L ACC, L DLPFC | Yes | No | Subdural hematoma after a fall |
| D5 | 06-021 | Li, BZD | Probably | L ACC, L DLPFC | No | No | Severe neck trauma with contusio cerebri and pneumonia |
| D6 | 06-075 | Li, Prednisolone | No | L ACC, L DLPFC | ND | No | Acute heart death |
| D7 | 07-060 | BZD | Yes | L ACC | ND | No | Cachexia, failure of renal functions |
| D8 | 07-076 | Mo, Car | ND | L DLPFC | No | No | ND |
| D9 | 97-058 | Parnate | Yes | L ACC, L DLPFC | No | No | Respiratory insufficiency, lung embolism, cordial decompensation |
| D10 | 99-118 | None | Yes | L DLPFC | No | No | Cardiac ischemia |
| D11 | 01-074 | SSRI | Yes | L ACC, R DLPFC | No | No | Pons hemorrhage |
| D12 | 02-051 | Hal | No | L ACC, L DLPFC | ND | No | Renal insufficiency |
| D13 | 06-011 | Hal, Dex | Yes | L ACC, L DLPFC | No | No | Legal euthanasia because of metastasized mamma carcinoma |
| D14 | 06-026 | BZD, Mo, Clozapine | Yes | L ACC, L DLPFC | No | No | Respiratory insufficiency |
| D15 | 07-033 | BZD, Levetiracetam, Mo | Yes | L ACC, L DLPFC | No | No | Multiple epileptic seizures |
| C1 | 00-067 | ND |  | L DLPFC |  |  | Massive lung emboli |
| C2 | 01-033 | Ipratropium, Mo |  | L DLPFC |  |  | Dehydration, adenocarcinoma, pneumonia |
| C3 | 01-086 | Mo |  | L DLPFC |  |  | Heart failure |
| C4 | 04-020 | Mo |  | L ACC |  |  | Pneumonia caused by aspiration |
| C5 | 04-049 | BDZ, TCA -only during the night to sleep for the last 10 days |  | L ACC, L DLPFC |  |  | Cachexia and uremia |
| C6 | 04-057 | Mo |  | L ACC, L DLPFC |  |  | Legal euthanasia because of metastasized cholangiocarcinoma |
| C7 | 05-017 | Mo |  | L ACC |  |  | Pneumonia, heart infarction, renal insufficiency |
| C8 | 05-019 | Mo, Hal, BZD |  | L ACC, L DLPFC |  |  | Bronchus carcinoma |
| C9 | 05-034 | ND |  | L DLPFC |  |  | Terminal congestive heart failure |
| C10 | 05-044 | Mo |  | L ACC, L DLPFC |  |  | Cachexia and dehydration |
| C11 | 05-068 | None |  | L ACC |  |  | Acute myocardial infarction |
| C12 | 05-073 | None |  | L ACC |  |  | Unknown |
| C13 | 06-037 | Testosterone, Methylphenidate |  | L DLPFC |  |  | Ruptured abdominal aorta aneurysm |
| C14 | 06-080 | BZD |  | L ACC |  |  | Died 2 days after a fall |
| C15 | 95-062 | None |  | L ACC |  |  | Renal insufficiency with metabolic acidosis and hyperpotassemia |
| C16 | 96-052 | None |  | L DLPFC |  |  | Cardiac arrest, probably due to tamponade |
| C17 | 97-039 | None |  | L DLPFC |  |  | Myocardial infarction |
| C18 | 97-043 | None |  | L ACC |  |  | Heart infarction |
| C19 | 97-143 | None |  | L ACC |  |  | Extensive metastases of adenocarcinoma of the prostate and diffuse tumor embolisms in both lungs, lung edema |
| C20 | 97-156 | None |  | L DLPFC |  |  | Septic shock, Metastasized pancreas carcinoma |
| C21 | 98-006 | None |  | L DLPFC |  |  | Cardiac arrest, sepsis |
| C22 | 99-111 | None |  | L DLPFC |  |  | Respiration insufficiency, cardial decompensation, peritonitis |

**Table S1 Continued**

Abbreviations: ACC, anterior cingulate cortex; BPD, bipolar disorder; BZD, benzodiazepine; Car, carbamazepine; CSF, cerebrospinal fluid; Dex, dexamethasone; DLPFC, dorsolateral prefrontal cortex; ECT, electro-convulsive treatment; F, female; Hal, haloperidol; L, left; Li, lithium; M, male; MDD, major depressive disorder; Mo, morphine; NBB, Netherlands Brain Bank; ND, no data; None, no medication; PMD, postmortem delay; R, right; RIN, RNA integrity number with 1 being the lowest and 10 being the highest quality of RNA; SSRI, selective serotonin reuptake inhibitor; TCA, tricyclic antidepressant; ZUC, zuclopenthixol.

^1^, Ventricular CSF pH correlates well with brain pH (Hardy et al., 1985) and is an indication of the brain RNA quality (Tomita et al., 2004).

**Table S2. P-values for matching of subjects with mood disorders and controls.**

| ACC | Ctr^BPD^ | BPD | Z | P (Ctr vs BPD) | Ctr^MDD^ | MDD | Z | P (Ctr vs MDD) |
| --- | --- | --- | --- | --- | --- | --- | --- | --- |
| Age^a^ (years, range) | 79 (68-89) | 78 (68-93) | -0.064 | 0.949 | 81 (56-96) | 70 (45-88) | -0.838 | 0.402 |
| Gender (M/F) | 5M/2F | 5M/2F | - | - | 4M/1F | 4M/1F | - | - |
| Race | 7W | 7W | - | - | 5W | 5W | - | - |
| PMD^a^ (hr:min, range) | 7:15 (5:00-10:20)) | 6:30 (5:15-16:46) | -0.128 | 0.898 | 6:05 (4:30-9:15) | 6:37 (4:20-7:15) | -0.104 | 0.917 |
| CTD^b^ (hh:mm, range) | 07:15 (02:00-21:30) | 10:15 (09:30-23:00) | - | 0.021^c^ | 13:27 (04:45-20:15) | 15:30 (02:30-21:15) | - | 0.085 |
| Ventricular CSF pH^a^ | 6.48 (5.8-7.08) | 6.375 (6.27-6.64) | -0.571 | 0.568 | 6.7 (6.22-7.16) | 6.5 (6.26-6.55) | -1.230 | 0.219 |
| Brain weight^a^ (gram, range) | 1356 (1125-1547) | 1260 (1143-1488) | -0.064 | 0.949 | 1400(1164-1568) | 1345 (1080-1427) | -0.522 | 0.602 |
| RIN^a^ | 7.5 (5.2-8.6) | 7.1 (6.3-7.7) | -0.704 | 0.481 | 7.5 (5.4-7.9) | 7.9 (7-8.1) | -0.841 | 0.401 |
| DLPFC | Ctr^BPD^ | BPD | Z | P (Ctr vs BPD) | Ctr^MDD^ | MDD | Z | P (Ctr vs MDD) |
| Age^a^ (years, range) | 74 (50-81) | 73 (68-90) | -0.089 | 0.929 | 87 (56-88) | 70(45-88) | -0.952 | 0.341 |
| Gender (M/F) | 6M/3F | 6M/3F | - | - | 4M/1F | 4M/1F | - | - |
| Race | 9W | 9W | - | - | 5W | 5W | - | - |
| PMD^a^ (hr:min, range) | 7:45 (2:40-24:45) | 6:30 (4:50-16:46) | -0.662 | 0.508 | 6:20 (4:00-14:00) | 6:37 (4:20-7:15) | -0.210 | 0.834 |
| CTD^b^ (hh:mm, range) | 08:30 (00:01-17:45) | 09:52 (02:45-23:15) | - | 0.986 | 03:05 (00:01-15:00) | 15:30 (02:30-21:15) | - | 0.237 |
| Ventricular CSF pH^a^ | 6.65 (5.8-7.16) | 6.36 (6.26-6.82) | -1.159 | 0.246 | 6.84 (6.18-7.39) | 6.5 (6.26-6.55) | -1.476 | 0.140 |
| Brain weight^a^ (gram, range) | 1312 (1125-1590) | 1231 (1143-1490) | -0.751 | 0.453 | 1323 (1054-1506) | 1345 (1080-1427) | -0.313 | 0.754 |
| RIN^a^ | 7.8 (5.7-8.5) | 7.2 (6.3-8.1) | -1.636 | 0.102 | 8.1 (6.9-8.5) | 8.0 (7.3-8.9) | -0.317 | 0.751 |

Abbreviations: ACC, anterior cingulate cortex; BPD, bipolar disorder; CSF, cerebrospinal fluid; CTD, clock time of death: Ctr, control subjects; DLPFC, dorsolateral prefrontal cortex; F, female; M, male; MDD, major depressive disorder; PMD, postmortem delay; RIN, RNA integrity number with 1 being the lowest and 10 being the highest quality of RNA; W, White Caucasian.

^a^, nonparametric Mann-Whitney U-test

^b^, Mardia-Watson-Wheeler test

^c^, This does not seem to influence our data since no day/night fluctuation were found in any gene in the ACC controls and there is no correlation between CTD and astrocyte-related gene expression we determined.

**Table. S3 Results of target genes changes in the ACC and DLPFC between mood disorder group and control group**

| **Genes** | **Median**  **(Range)** | | | | **Fold changes** | | **p values** | |
| --- | --- | --- | --- | --- | --- | --- | --- | --- |
| **ACC** | **Ctr^BPD^** | **BPD** | **Ctr^MDD^** | **MDD** | **BPD/Ctr^BPD^** | **MDD/Ctr^MDD^** | **BDvsCtr^BPD^** | **MDDvsCtr^MDD^** |
| GFAP | 2.71399  (1.24779-3.06039) | 3.56122  (2.34038-8.87344) | 5.48832  (1.54368-8.29628) | 1.68422  (0.87390-4.58817) | 1.31217 | 0.30687 | **0.018↑** | 0.076 |
| vimentin | 0.44942  (0.27183-0.61927) | 0.46147  (0.34095-0.76873) | 0.43164  (0.23770-1.92324) | 0.33034  (0.22324-0.95425) | 1.02681 | 0.76531 | 0.482 | 0.347 |
| Synemin-α | 0.14797  (0.11308-0.25652) | 0.17112  (0.10194-0.29176) | 0.15337  (0.07964-0.22212) | 0.13770  (0.08573-0.19070) | 1.15645 | 0.89783 | 0.848 | 0.465 |
| Synemin-β | 0.05444  (0.03994-0.09308) | 0.06562  (0.05231-0.16399) | 0.07154  (0.04105-0.10052) | 0.04722  (0.03541-0.05250) | 1.20536 | 0.66005 | 0.085 | 0.076 |
| nestin | 0.04393  (0.02244-0.06494) | 0.03768  (0.02166-0.10136) | 0.03273  (0.02575-0.08992) | 0.02388  (0.01795-0.03702) | 0.85773 | 0.72961 | 0.749 | 0.175 |

| **Genes** | **Median**  **(Range)** | | | | **Fold changes** | | **p values** | |
| --- | --- | --- | --- | --- | --- | --- | --- | --- |
| **DLPFC** | **Ctr^BPD^** | **BPD** | **Ctr^MDD^** | **MDD** | **BPD/Ctr^BPD^** | **MDD/Ctr^MDD^** | **BDvsCtr^BPD^** | **MDDvsCtr^MDD^** |
| GFAP | 0.93841  (0.60949-1.86520) | 1.45328  (0.51257-4.33688) | 2.10110  (0.68420-2.45717) | 1.11562  (0.68123-1.65308) | 1.54866 | 0.53097 | 0.122 | 0.251 |
| vimentin | 0.26168  (0.14818-0.77349) | 0.31279  (0.16789-0.62557) | 0.32094  (0.19421-0.52412) | 0.37242  (0.19019-0.87357) | 1.19531 | 1.16040 | 0.691 | 0.602 |
| Synemin-α | 0.11782  (0.07389-0.20492) | 0.14880  (0.09341-0.22142) | 0.15498  (0.12451-0.23922) | 0.13227  (0.09729-0.14685) | 1.26294 | 0.85346 | 0.270 | 0.175 |
| Synemin-β | 0.04445  (0.02119-0.07834) | 0.04612  (0.02751-0.09706) | 0.04233  (0.02630-0.08287) | 0.04149  (0.03230-0.05677) | 1.03766 | 0.98016 | 0.825 | 0.917 |
| nestin | 0.05480  (0.03006-0.09325) | 0.05687  (0.03054-0.11513) | 0.03171  (0.02567-0.09398) | 0.04880  (0.03825-0.07169) | 1.03783 | 1.53895 | 0.895 | 0.117 |

Abbreviations: ACC, anterior cingulate cortex; BPD, bipolar disorder; Ctr, control, DLPFC, dorsolateral prefrontal cortex; GFAP, glial fibrillary acidic protein; MDD, major depressive disorder

↑ indicates a significant increase (p< 0.05) for alterations

**Table. S4 Results of correlation between the GFAP mRNA expression level and other parameters in the ACC in the control group and mood disorder group**

| ACC | GFAP mRNA | | |
| --- | --- | --- | --- |
|  | Control / 12 subjects | BPD / 7 subjects | MDD / 5 subjects |
| vimentin mRNA | **Spearman’s rho=0.692, p=0.013** | **Spearman’s rho=0.893, p=0.007** | Spearman’s rho=-0.700, p=0.188 |
| synemin-α mRNA | Spearman’s rho=0.503, p=0.095 | Spearman’s rho=0.429, p=0.337 | Spearman’s rho=0.800, p=0.104 |
| synemin-β mRNA | **Spearman’s rho=0.888, p=0.001** | **Spearman’s rho=0.857, p=0.014** | Spearman’s rho=-0.600, p=0.285 |
| nestin mRNA | Spearman’s rho=0.503, p=0.095 | Spearman’s rho=0.714, p=0.071 | Spearman’s rho=0.700, p=0.188 |
| GFAP-ir astrocytes density | Spearman’s rho=0.350, p=0.265 | Spearman’s rho=0.321, p=0.482 | Spearman’s rho=0.624, p=0.300 |
| GFAP-ir astrocytes area fraction | Spearman’s rho=0.483, p=0.112 | Spearman’s rho=0.357, p=0.432 | Spearman’s rho=0.624, p=0.300 |
| GFAP-ir astrocytes IOD | **Spearman’s rho=0.650, p=0.022** | Spearman’s rho=0.214, p=0.645 | Spearman’s rho=0.624, p=0.300 |

Abbreviations: ACC, anterior cingulate cortex; BPD, bipolar disorder; GFAP, glial fibrillary acidic protein; GFAP-ir, GFAP-immunoreactive; MDD, major depressive disorder

**Table S5. Table of sequence of primers, gene bank accession numbers and the length of amplified product for the target genes and reference genes**

| Gene | Primer sequences | Accession numbers | Amplicon length(bp) |
| --- | --- | --- | --- |
| GFAP | GGGCATGACTTTGTCCCATTT | NM_002055 | 118 |
|  | ACGTGTGGCAGTGGCTTACTG |  |  |
| Synemin-α | TGCAGGGAAGGTTGGTGATTA | NM_145728 | 104 |
|  | TTTGCCCACTGAACCCTTCTT |  |  |
| Synemin-β | ATAAAACTCGGCCCCTCTGAAG | NM_015286 | 103 |
|  | GGATCGCCTCTACGTTACTCACAT |  |  |
| Vimentin | CGTACGTCAGCAATATGAAAGTGTG | NM_003380 | 87 |
|  | TCAGAGAGGTCAGCAAACTTGGA |  |  |
| Nestin | ATCTAAACAGGAAGGAAATCCAGG | NM_006617 | 123 |
|  | CTAGTGTCTCATGGCTCTGGTTTT |  |  |
| ACT β | CCCAGCCATGTACGTTGCTA | NM_001101 | 65 |
|  | TCACCGGAGTCCATCACGAT |  |  |
| GAPDH | CAAATTCCATGGCACCGTC | NM_002046 | 62 |
|  | TCTCGCTCCTGGAAGATGGT |  |  |
| HPRT1 | GGACAGGACTGAACGTCTTGC | NM_000194 | 88 |
|  | ATAGCCCCCCTTGAGCACAC |  |  |
| TUB α | CTTTGAGCCAGCCAACCAGA | NM_006082 | 72 |
|  | GTACAACAGGCAGCAAGCCAT |  |  |
| UBC | GCTGCTCATAAGACTCGGCC | NM_021009 | 70 |
|  | GTCACCCAAGTCCCGTCCTA |  |  |
| HMBS | GATCCCGAGACTCTGCTTCG | NM_001024382 | 70 |
|  | ACACTGCAGCCTCCTTCCAG |  |  |
| TUB β4 | GGGCCAAGTTTTGGGAGGT | NM_006087 | 71 |
|  | CACTGTCCCCATGGTATGTGC |  |  |

Abbreviations: ACT β, actin-β; GAPDH, glyceraldehydes-3-phosphate dehydrogenase; GFAP, glial fibrillary acidic protein; HMBS, hydroxymethylbilane synthase; HPRT1,hypoxanthine phosphoribosyltransferase 1; TUB α, tubulin-α; TUB β4, tubulin-β4; UBC, ubiquitin C.

**Figure S1**


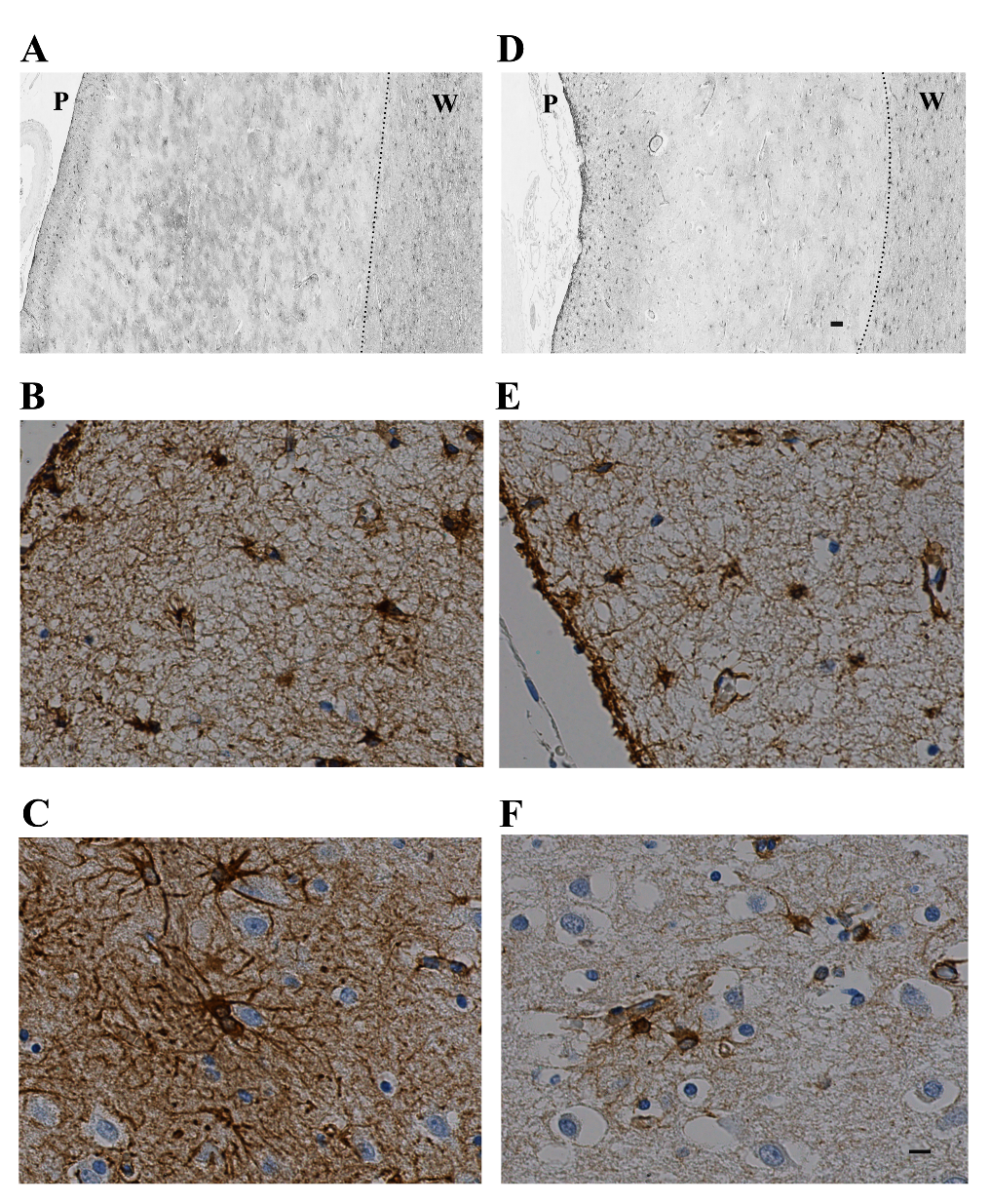


Legend: Representative image of GFAP-ir astrocytes in the ACC of a control subject (NBB No. 05-068) with low magnification image (A) from pia mater (P) to white matter (W) covering all cortical layers and high magnification image in the layer I (B) and layer III (C). Representative image of GFAP-ir astrocytes in the ACC of a BPD patient (NBB No. 00-074) with low magnification image (D) from pia mater (P) to white matter (W) covering all cortical layers and high magnification image in the layer I (E) and layer III (F). Note the reduction of GFAP-positive astrocyte processes and remodeling of the astrocyte network in BPD. Scale bar = 100 μm in D; Scale bar = 10 μm in F. Dash line between grey matter and white matter.
